# The clinical impact of phase offset errors and different correction methods in cardiovascular magnetic resonance phase contrast imaging: a multi-scanner study

**DOI:** 10.1186/s12968-020-00659-3

**Published:** 2020-09-17

**Authors:** Savine C. S. Minderhoud, Nikki van der Velde, Jolanda J. Wentzel, Rob J. van der Geest, Mohammed Attrach, Piotr A. Wielopolski, Ricardo P. J. Budde, Willem A. Helbing, Jolien W. Roos-Hesselink, Alexander Hirsch

**Affiliations:** 1 Department of Cardiology, Erasmus Medical Center, University Medical Center Rotterdam, P.O. Box 2040, Room Rg-419, Rotterdam, 3000 CA the Netherlands; 2 Department of Radiology and Nuclear Medicine, Erasmus Medical Center, University Medical Center Rotterdam, Rotterdam, The Netherlands; 3 Department of Radiology, Division of Image Processing, Leiden University Medical Center, Leiden, The Netherlands; 4 Division of Pediatric Cardiology, Department of Pediatrics, Erasmus Medical Center, University Medical Center Rotterdam, Rotterdam, The Netherlands

**Keywords:** Flow quantification, Cardiovascular magnetic resonance imaging, Phase contrast velocity imaging, Phase offset error

## Abstract

**Background:**

Cardiovascular magnetic resonance (CMR) phase contrast (PC) flow measurements suffer from phase offset errors. Background subtraction based on stationary phantom measurements can most reliably be used to overcome this inaccuracy. Stationary tissue correction is an alternative and does not require additional phantom scanning. The aim of this study was 1) to compare measurements with and without stationary tissue correction to phantom corrected measurements on different GE Healthcare CMR scanners using different software packages and 2) to evaluate the clinical implications of these methods.

**Methods:**

CMR PC imaging of both the aortic and pulmonary artery flow was performed in patients on three different 1.5 T CMR scanners (GE Healthcare) using identical scan parameters. Uncorrected, first, second and third order stationary tissue corrected flow measurement were compared to phantom corrected flow measurements, our reference method, using Medis QFlow, Circle cvi42 and MASS software. The optimal (optimized) stationary tissue order was determined per scanner and software program. Velocity offsets, net flow, clinically significant difference (deviation > 10% net flow), and regurgitation severity were assessed.

**Results:**

Data from 175 patients (28 (17–38) years) were included, of which 84% had congenital heart disease. First, second and third order and optimized stationary tissue correction did not improve the velocity offsets and net flow measurements. Uncorrected measurements resulted in the least clinically significant differences in net flow compared to phantom corrected data. Optimized stationary tissue correction per scanner and software program resulted in net flow differences (> 10%) in 19% (MASS) and 30% (Circle cvi42) of all measurements compared to 18% (MASS) and 23% (Circle cvi42) with no correction. Compared to phantom correction, regurgitation reclassification was the least common using uncorrected data. One CMR scanner performed worse and significant net flow differences of > 10% were present both with and without stationary tissue correction in more than 30% of all measurements.

**Conclusion:**

Phase offset errors had a significant impact on net flow quantification, regurgitation assessment and varied greatly between CMR scanners. Background phase correction using stationary tissue correction worsened accuracy compared to no correction on three GE Healthcare CMR scanners. Therefore, careful assessment of phase offset errors at each individual scanner is essential to determine whether routine use of phantom correction is necessary.

**Trial registration:**

Observational Study

## Introduction

Cardiovascular magnetic resonance (CMR) 2D-phase contrast (PC) imaging is used to quantify blood flow in the great arteries. Spins moving along a magnetic field gradient acquire a shift in their phase of rotation in comparison to stationary spins. This phase shift is proportional to the velocity of blood [1]. The net flow is calculated by integration of the velocity over time per heartbeat and taking a second integral over the cross-sectional area of the vessel. Flow quantification is helpful for assessment of valvular regurgitant fraction and shunts, and often have important therapeutic consequences in deciding whether valvular surgery is necessary [2].

Common measurement errors of PC imaging include mismatched encoding velocity, deviation of the imaging plane, inadequate temporal resolution and spatial resolution and phase offset errors [1]. These errors can be overcome during scanning when the scanning operator is appropriately trained, except for phase offset errors [3, 4]. Phase offset errors are errors caused by non-compensated eddy-current-induced fields and concomitant gradient terms [1, 3]. Phase offset errors result in velocity offsets, meaning the measured velocities deviate from the actual velocities. The extent of this error depends on gradient imbalance due to eddy currents, Maxwell terms and gradient field nonlinearity [5–8]. A general correction for phase offset errors is not possible as the severity of phase offset errors and influence on uncorrected flow measurements vary greatly per specific acquisition and across CMR systems [9].

Reliable background offset correction is necessary. Stationary tissue correction is a method available in many (commercial) software applications. The velocity offset in stationary tissue is used to estimate the velocity offset at the vessel of interest. Most commonly, interpolation methods which assume a linear variation over the field-of-view are used. This method does not require acquisition of additional CMR sequences. However, stationary tissue is most frequently not directly located next to the vessel of interest and the magnitude of phase offset errors varies spatially over the imaging plane. With the offset information in stationary tissue, the magnitude of phase offset errors is estimated at the vessel of interest. Phantom correction is another approach to correct flow measurements for phase offset errors. With flow measurements in a stationary phantom, the magnitude of the phase offset errors at the location of the vessel of interest is assessed. This method assumes temporal stability of the phase offset errors and requires extra acquisitions and time, as every individual acquisition needs its own stationary phantom acquisition.

Recently, stationary tissue correction has been shown to reduce phase offset errors to minimal and clinically acceptable differences in small groups of patients with efficacy comparable to phantom measurements [10]. Therefore, the purpose of this study was 1) to compare stationary tissue corrected and uncorrected flow measurements with phantom corrected flow measurements in a large group of patients in daily clinical practice, 2) to assess the variation of phase offset errors across different CMR systems and 3) to investigate the impact of phase offset errors on regurgitation severity indexing.

## Methods

### Study population

In this retrospective study, all consecutive patients and healthy volunteers that underwent a CMR on one of our three clinical 1.5 T CMR systems used for cardiac scanning were screened. The inclusion criteria were availability of 2D-PC images with both through-plane flow of the aorta and main pulmonary artery and concomitant stationary phantom acquisitions. Both pediatric and adult patients were included as well as patients with and without shunt. There was no selection in referral to a specific scanner except that the majority of pediatric patients were scanned on one of the three scanners because of its location in our children’s hospital. Only patients with mechanical aortic or pulmonary valves were excluded due to potential imaging artifacts. Of each patient, age, sex, biometric data, diagnosis, presence of shunt lesions, valve type, and valvular disease were collected. Since this is a purely observational and retrospective study, the need for ethics committee approval was waived by the institutional review board (MEC-2019-0155). All healthy subjects were prospectively recruited and provided informed consent (MEC-2014-096).

### CMR protocol

CMR imaging was performed on the following three different clinical 1.5 T CMR scanners from General Electric Healthcare (Milwaukee, Wisconsin, USA): Signa Artist with software version DV26.0 (CMR-1), Discovery MR450 with DV25.0 (CMR-2), and Signa Explorer with DV25.0 (CMR-3). Characteristics of the different systems are shown in Additional file 1. Included patients had undergone a CMR examination with at least PC and cine imaging. Through plane 2D PC flow measurements of the aorta and pulmonary artery were performed during end-expiratory breath-hold using retrospective electrocardiographic (ECG) gating. The imaging planes were planned perpendicular to the great vessels. Aortic PC imaging was scanned at the aortic valve and pulmonary PC imaging was scanned approximately 1 cm above the valve proximal to the pulmonary bifurcation. The same sequence parameters were used on all three scanners: field-of-view (FOV) 31–38 cm, phase FOV 75–100%, slice thickness 7 mm, matrix size 192 × 160, flip angle 20^°^, echo time 3.4 ms (1.8–3.9), repetition time 5.8 ms (4.7–6.5), ASSET 1.5, views per segment 4 to 6 based on patients’ heart rate and 30 reconstructed cardiac phases. Standard velocity encoding (VENC) value was set at 180 cm/s, however, increased in gradual steps up to 500 cm/s if necessary, and flow compensation was used. Phase errors due to Maxwell/concomitant gradient terms were corrected within the image reconstruction and flow optimization was turned on to limit gradient slew rates in order to minimize eddy currents as advised by the vendor [10]. During PC acquisition, the structure of interest was aimed to be located in the magnetic isocenter and local shimming was used.

Furthermore, cine imaging was performed using a breath-hold segmented, balanced steady-state free precession sequence with a slice thickness of 6 mm and 4 mm interslice gap. Long and short axis images were made so that the whole left ventricle (LV) and right ventricle (RV) were covered from basis to apex.

Directly after the CMR examination, the exact same PC sequence parameters were used to scan a static gel phantom. The patient was positioned next to the scanner, still connected to the ECG recording and phantom acquisition was triggered based on the patients’ heart rate. Phantoms consisted of a 10 L paraben (C_10_H_12_O_3_) gelatin gel with 50 mL Gadovist. This object was positioned at the identical location on the table where the heart was located.

### CMR analysis

Currently, multiple software programs are available to analyze flow acquisitions. Medis software (QMass software version 8.1 and QFlow software version 2.3, Medis, Leiden, The Netherlands), Circle Cardiovascular Imaging (cvi42 version 5.11.2, Circle Cardiovascular Imaging, Calgary, Canada) and MASS research software (Version 2016EXP, Leiden University Medical Centre, Leiden, The Netherlands) were used to analyze the CMR images to assess also variability between software programs.

**Fig. 1:**
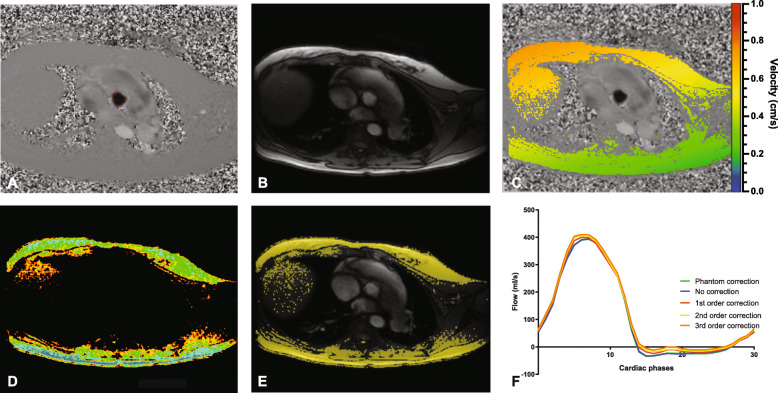
Example of phase contrast cardiovascular magnetic resonance (CMR) with **a**) Velocity image **b**) Magnitude image **c**) Stationary tissue detection in Medis QFlow **d**) Stationary tissue detection in MASS **e**) Stationary tissue detection in Circle cvi42 **f**) Examples of flow curves during 1 cardiac cycle measured with Circle cvi42 and shown for all different correction methods

For PC velocity analysis, the aorta and pulmonary artery were manually delineated in at least one cardiac phase (Fig. 1a and b). Automatic border detection was used for the other cardiac phases. These contours were reviewed and adapted accordingly for each cardiac phase. The same contours were used for analysis in MASS and Medis QFlow. For the analysis in Circle cvi42, separate contours were drawn. The net flow per cardiac phase was calculated by integration of the velocity per cardiac phase and taking a second integral over the vessel area.

Net flow was defined as total forward flow minus total regurgitation flow over 30 cardiac phases, which equals one heartbeat. This net flow was measured in mL per heartbeat and, subsequently, corrected for body surface area. Regurgitation fraction was calculated by dividing the regurgitation flow by the forward flow × 100%. Aortic valve regurgitation was graded as none (< 5%), mild (5–20%), moderate (20–33%) and severe (> 33%) [11]. Pulmonary regurgitation was defined as none (< 5%), mild (5–20%), moderate (20–40%) and severe (> 40%) [12]. Aortic and pulmonary valve stenosis were defined as a peak systolic velocity of ≥3 m/s. Q_p_/Q_s_ ratio was defined as pulmonary flow divided by systemic flow and a shunt lesion was defined as Q_p_/Q_s_ > 1.5 in phantom corrected flow measurements [13].

Within each software, flow measurements were analyzed in three manners: 1) uncorrected flow measurements, 2) stationary tissue corrected flow measurements (first order correction in all three software programs and second and third order correction in MASS and Circle cvi42), and 3) phantom corrected flow measurements.

For stationary tissue correction, areas of phase wrapping and cardiac structures were excluded. Within the remaining region, in Medis QFlow and Circle cvi42 25% and respectively 15% of all the pixels with the lowest variation in velocity during the cardiac cycle were regarded as stationary pixels (Fig. 1c and e). In MASS, of each pixel the standard deviation of the velocity over the cardiac cycle was calculated and pixels with a standard deviation < 2.5 cm/s were considered as stationary tissue (Fig. 1d). Based on these pixels, a linear (first order) and tilted and curved (second and third order) interpolation correction plane was made over the field-of-view. This plane allowed to estimate phase offset error in nonstationary pixels, i.e. the area within the drawn contours [3]. Subtracting the fitted surface from the phase image, the stationary tissue corrected results were obtained.

The phantom corrected blood flow was determined by subtraction of the phantom flow measurement from the initial flow measurement on a pixel by pixel basis. Phantom corrected flow measurements were used as reference standard. Net flow, regurgitation fraction, and Q_p_/Q_s_ ratio of uncorrected and stationary tissue corrected measurements were compared to phantom corrected flow measurements (Fig. 1f). The difference and absolute difference (regardless of over- or underestimation) in net flow were calculated in percentages. Differences of more than 10% in net flow were considered as clinically significant. Changes in regurgitation severity grading were assessed comparing uncorrected and stationary tissue corrected flow measurements with phantom corrected flow measurements.

Finally, the contours of the vessel of interest were imported onto the phantom images. Herewith, the initial velocity offset was determined before any correction method was applied. Also, the velocity offset after stationary correction was determined.

On the cine short-axis stack LV and RV endocardial contours were manually traced in end-diastole and end-systole according to Society for Cardiovascular Magnetic Resonance guidelines on CMR image post-processing [14]. Papillary muscles and trabeculations were excluded from the endocardial contours and LV and RV end-diastolic volume (EDV), end-systolic volume (ESV), and ejection fraction (EF) were determined (analysis performed with Medis QMass).

### Phantom accuracy

Phantom corrected measurements were used as a reference. This method assumes temporal stability of the velocity offset over time [15]. Although we performed the acquisition of the static phantom with identical slice orientation and parameters directly after the examination using the heartbeat of the patients, the validity and temporal stability of phantom correction method was tested in all studies with a similar method as described by Hofman et al. [10]. In short, a region of interest was drawn in stationary tissue at the anterior thorax wall in the PC image. This region-of-interest was copied onto the corresponding phantom image and checked if the region was completely covered by phantom. Average velocity offset within this region over the cardiac cycle in the in-vivo scan was compared to the velocity offset in the phantom scan (measured with Medis QFlow). Only if the agreement between these measurements was within 0.6 cm/s, the study was included in our analysis [9].

### Statistics

Continuous values were expressed as mean with standard deviation or as median with a range or interquartile (IQR) range in cases of skewed distributions. Categorical data were presented as frequencies and percentages. Differences in velocity offset and net flow were tested for significance using a paired T-test if normally distributed and with a Wilcoxon signed rank test if not normally distributed. Differences in net flow were also visualized using root mean square (RMS) values. This gives a better indication of the difference in the individual patient independently of over- or underestimation. Changes in clinically significant differences were tested between correction methods with a McNemar’s test and between scanners with a Chi-square test. Based on the number of clinically significant differences, absolute velocity offset, standard deviation of the velocity offset and RMS, the optimal stationary tissue order per scanner and per software program was determined. Linear regression was used to investigate the association of uncorrected and stationary tissue corrected measurements of net flow and Q_p_/Q_s_ ratios with phantom corrected net flow and Q_p_/Q_s_ ratios. Pearson correlations were calculated. A weighted kappa was used to assess differences in regurgitation severity classification between correction methods and phantom correction. Linear regression was performed to identify the dependence of velocity offset on velocity encoding (VENC), heart rate, vessel size, maximum blood flow velocity, vessel location (aorta or main pulmonary) and scanner. Forward method was used for multivariate linear regression, in order to identify independent predictors for phase offset errors. Variables were entered in the model if the *p*-values was less than 0.20.

Statistical analyses were conducted using SPSS (version 25.0, Statistical Package for the Social Sciences, International Business Machines, Inc., Armonk, New York, USA) and R Statistical Software (version 3.6.1, R Foundation for Statistical Computing, Vienna, Austria). *P*-values of < 0.05 were considered statistically significant.

## Results

### Phantom accuracy and study population

Initially 177 patients were included in this study including 354 flow acquisitions. No studies were excluded due to too much wraparound. In 19 of the 175 studies, small areas of spatial wraparound were present and these areas were manually excluded. First the accuracy of the phantom correction method was tested. Median difference between in-vivo stationary tissue measurements and phantom measurements was − 0.1 (− 0.3 to 0.2) cm/s. The assumption of stability of the velocity offset over time (> 0.6 cm/s) was violated in two aortic flow examinations and six pulmonary flow examinations within three patients scanned on CMR-2 and three scanned on CMR-3. Therefore, these eight studies were excluded from further analysis. The final analysis consisted of 175 patients with 346 flow acquisitions.

Of the 175 included patients 76 were scanned on CMR-1, 48 on CMR-2, and 51 on CMR-3. Patient characteristics are summarized in Table 1. Patients were 28 (17–38) years old, and 102 (58%) were male. Of the included patients, 147 (84%) had a congenital heart defect, of which tetralogy of Fallot was the most prevalent diagnosis (60 patients (34%)). Patients scanned on CMR-3 were younger and, therefore, the dimensions of the aorta and main pulmonary artery were smaller.

**Table 1:** Baseline table

	CMR-1 (***n*** = 76)	CMR-2 (***n*** = 48)	CMR-3 (***n*** = 51)	Overall (***n*** = 175)
**Age**	31 (25–42)	30 (20–40)	15 (11–21)	28 (17–38)
Age < 18 years	4 (5%)	5 (10%)	35 (69%)	44 (25%)
Range	12–68	16–66	5–64	5–68
**Male**	41 (54%)	32 (67%)	29 (57%)	102 (58%)
**Weight (kg)**	76 (65–86)	70 (56–80)	55 (40–70)	69 (55–81)
**Height (cm)**	174 (165–182)	174 (164–182)	162 (150–175)	172 (159–180)
**Body surface area (BSA) (m**^**2**^**)**	1.9 (1.7–2.1)	1.8 (1.6–2.0)	1.5 (1.3–1.9)	1.8 (1.6–2.0)
**Heart rate (beats/min)**	68 ± 12	71 ± 12	72 ± 13	70 ± 13
**Diagnosis**				
Tetralogy of Fallot, PA + VSD	20 (26%)	15 (31%)	25 (49%)	60 (34%)
ASD	2 (3%)	1 (2%)	5 (10%)	8 (5%)
VSD	0 (0%)	4 (8%)	5 (10%)	9 (5%)
TGA	6 (8%)	5 (10%)	2 (4%)	13 (7%)
Valvular disease	28 (37%)	12 (25%)	12 (24%)	52 (30%)
Turner syndrome	7 (9%)	5 (10%)	0 (0%)	12 (7%)
Other	4 (5%)	5 (10%)	2 (4%)	11 (6%)
Healthy volunteer	9 (12%)	1 (2%)	0 (0%)	10 (6%)
**Cardiac morphology & function**				
Shunt lesions	1 (1%)	3 (6%)	3 (6%)	7 (4%)
LV ejection fraction (%)^a^	55 (51–59)	55 (47–61)	57 (52–64)	55 (50–61)
LV end-diastolic volume (ml)^a^	155 (130–200)	164 (142–199)	144 (102–177)	155 (124–194)
LV end-systolic volume (ml)^a^	72 (57–89)	76 (58–100)	59 (40–86)	71 (53–89)
RV ejection fraction (%)^a^	52 (47–57)	52 (45–56)	53 (48–59)	52 (47–57)
RV end-diastolic volume (ml)^a^	190 (155–231)	216 (174–279)	170 (131–234)	192 (150–248)
RV end-systolic volume (ml)^a^	93 (67–120)	107 (79–137)	79 (54–117)	95 (63–123)
**Aortic valve**				
Regurgitation	9 (12%)	8 (17%)	3 (6%)	20 (11%)
Stenosis	2 (3%)	2 (4%)	0 (0%)	4 (2%)
Bicuspid	14 (18%)	3 (6%)	2 (4%)	19 (11%)
Biological valve	4 (5%)	0 (0%)	0 (0%)	4 (2%)
**Pulmonary valve**				
Regurgitation	25 (33%)	21 (45%)	23 (48%)	69 (40%)
Stenosis	6 (8%)	3 (6%)	2 (4%)	11 (6%)
Biological valve	10 (13%)	6 (13%)	7 (15%)	23 (13%)
**Vessel size**				
Aorta (cm^2^)	5.5 (4.3–7.7)	5.9 (4.7–9.0)	4.7 (3.8–6.1)	5.3 (4.3–7.6)
Main pulmonary artery (cm^2^)	6.2 (4.9–7.3)	5.8 (4.7–7.5)	4.9 (4.0–5.8)	5.6 (4.5–7.2)

Values are presented as numbers (percentage), mean ± SD or median (interquartile range)

*ASD* Atrium septal defect, *AVSD* Atrioventricular septal defect, *PA* Pulmonary atresia, *TGA* Transposition of the great arteries, *VSD* Ventricular septal defect

^a^ Data were missing in four patients

### Velocity offset

**Fig. 2:**
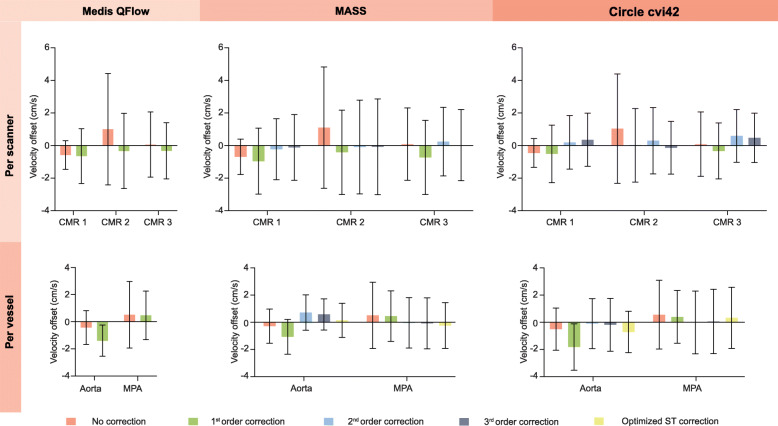
Velocity offset (cm/s) with no correction, first, second, third order and optimized stationary tissue correction measured in three different software packages. Top row stratified by scanner and bottom row stratified by vessel of interest (aorta or main pulmonary artery (MPA))

Before correction, velocity offset was 0.0 ± 2.0 cm/s (Medis QFlow), 0.1 ± 2.0 cm/s (MASS), and 0.0 ± 2.2 cm/s (Circle cvi42) depending on the software program used. The velocity offset using different software programs and for different order stationary tissue correction methods is shown in Table 2 and Fig. 2. Overall, interpolation-based correction did not improve the velocity offsets and the absolute velocity offset remained large. With regard to the different CMR systems, CMR-2 had a larger velocity offset than the other two systems and there was a larger range in over- and underestimation (Fig. 2, top row). The differences between the aortic and pulmonary measurements are depicted in Fig. 2, bottom row. Especially, first order correction did result in larger velocity offset in the aortic images. The optimized stationary tissue correction per scanner and software was: (1) second order for CMR-1, third order for CMR-2 and first order for CMR-3 for analyses in MASS and (2) second order for CMR-1, first order for CMR-2 and third order for CMR-3 for analyses in Circle cvi42. For further analyses, these optimized interpolation orders were used as a separate stationary tissue correction method and compared to uncorrected and phantom corrected measurements.

**Table 2:** Magnitude of flow change with and without offset correction

	No correction	Optimized ST correction	1st order correction	2nd order correction	3rd order correction	P-value῀ no vs. opt.	P-value ‡ no vs. 1st	P-value ‖ no vs. 2nd	P-value $ no vs 3rd
**Medis QFlow**									
Net flow (ml/m^2^)^a^	48 ± 13	–	47 ± 13	–	–	–	–	–	–
Velocity offset (cm/s)	0.0 ± 2.0	–	− 0.5 ± 1.8	–	–	–	< 0.001	–	–
Absolute velocity offset (cm/s)	1.0 ± 1.7	–	1.3 ± 1.2	–	–	–	< 0.001	–	–
Difference in net flow with phantom correction (%)^b^									
Difference	0.2 ± 15.4	–	−3.2 ± 12.8	–	–	–	< 0.001	–	–
Absolute difference	3.3 (1.2 to 7.3)	–	6.1 (3.0 to 11.2)	–	–	–	< 0.001	–	–
Range of difference	− 78 to 155	–	−66 to 74	–	–	–	–	–	–
Clinically significant differences (> 10%)	65 (19%)	–	103 (30%)	–	–	–	< 0.001	–	–
**MASS**									
Net flow (ml/m^2^)^a^	48 ± 13	48 ± 12	47 ± 13	49 ± 13	48 ± 12	–	–	–	–
Velocity offset (cm/s)	0.1 ± 2.0	0.0 ± 1.5	−0.3 ± 1.8	0.3 ± 1.6	0.3 ± 1.6	0.247	< 0.001	0.082	0.276
Absolute velocity offset (cm/s)	1.0 ± 1.7	0.9 ± 1.2	1.3 ± 1.3	1.1 ± 1.2	1.1 ± 1.1	0.418	< 0.001	0.013	0.003
Difference in net flow with phantom correction (%)^b^									
Difference	0.8 ± 15.6	−0.1 ± 11.1	−2.0 ± 13.0	2.7 ± 13.4	1.8 ± 12.2	0.379	< 0.001	0.060	0.281
Absolute difference	3.2 (1.3 to 7.2)	3.7 (1.5 to 8.5)	5.6 (2.4 to 10.4)	4.3 (1.6 to 9.3)	4.6 (2.2 to 9.2)	0.342	< 0.001	0.015	0.004
Range of difference	−78 to 161	−35 to 101	−65 to 69	−58 to 101	− 37 to 120	–	–	–	–
Clinically significant differences (> 10%)	62 (18%)	67 (19%)	93 (27%)	74 (21%)	79 (23%)	< 0.001	< 0.001	< 0.001	< 0.001
**Circle cvi42**									
Net flow (ml/m^2^)^a^	47 ± 13	47 ± 13	45 ± 12	47 ± 13	47 ± 13	–	–	–	–
Velocity offset (cm/s)	0.0 ± 2.2	−0.2 ± 2.0	− 0.7 ± 2.2	− 0.1 ± 2.1	− 0.1 ± 2.2	0.063	< 0.001	0.607	0.566
Absolute velocity offset (cm/s)	1.2 ± 1.9	1.4 ± 1.5	1.7 ± 1.6	1.4 ± 1.6	1.5 ± 1.6	0.006	< 0.001	0.014	< 0.001
Difference in net flow with phantom correction (%)^b^									
Difference	0.0 ± 16.0	−1.8 ± 14.3	−4.8 ± 15.0	− 0.5 ± 15.2	−0.7 ± 15.5	0.046	< 0.001	0.640	0.563
Absolute difference	4.1 (1.4 to 9.2)	6.0 (2.2 to 11.9)	8.0 (4.3 to 13.6)	5.3 (2.2 to 12.1)	6.1 (2.2 to 13.2)	0.005	< 0.001	0.011	< 0.001
Range of difference	−93 to 131	−62 to 59	−66 to 59	− 85 to 71	−80 to 75	–	–	–	–
Clinically significant differences (> 10%)	80 (23%)	103 (30%)	137 (40%)	107 (31%)	115 (33%)	< 0.001	< 0.001	< 0.001	< 0.001

Values are presented as mean ± standard deviation, median (interquartile range), range minimum to maximum or number (percentage)

*Opt.* Optimized stationary tissue (ST) correction

**῀** *p*-values no correction versus optimized ST correction, ‡ *p*-values no correction versus first order correction, ‖ *p*-values no correction versus second order correction, $ *p*-values no correction versus third order correction

^a^ Indicates net flow per heartbeat, with phantom correction the mean net flow was 48 ± 11 ml/ m^2^ in all software packages, ^b^ Minus indicates flow was lower compared to phantom flow measurements

### Net flow quantification

**Fig. 3:**
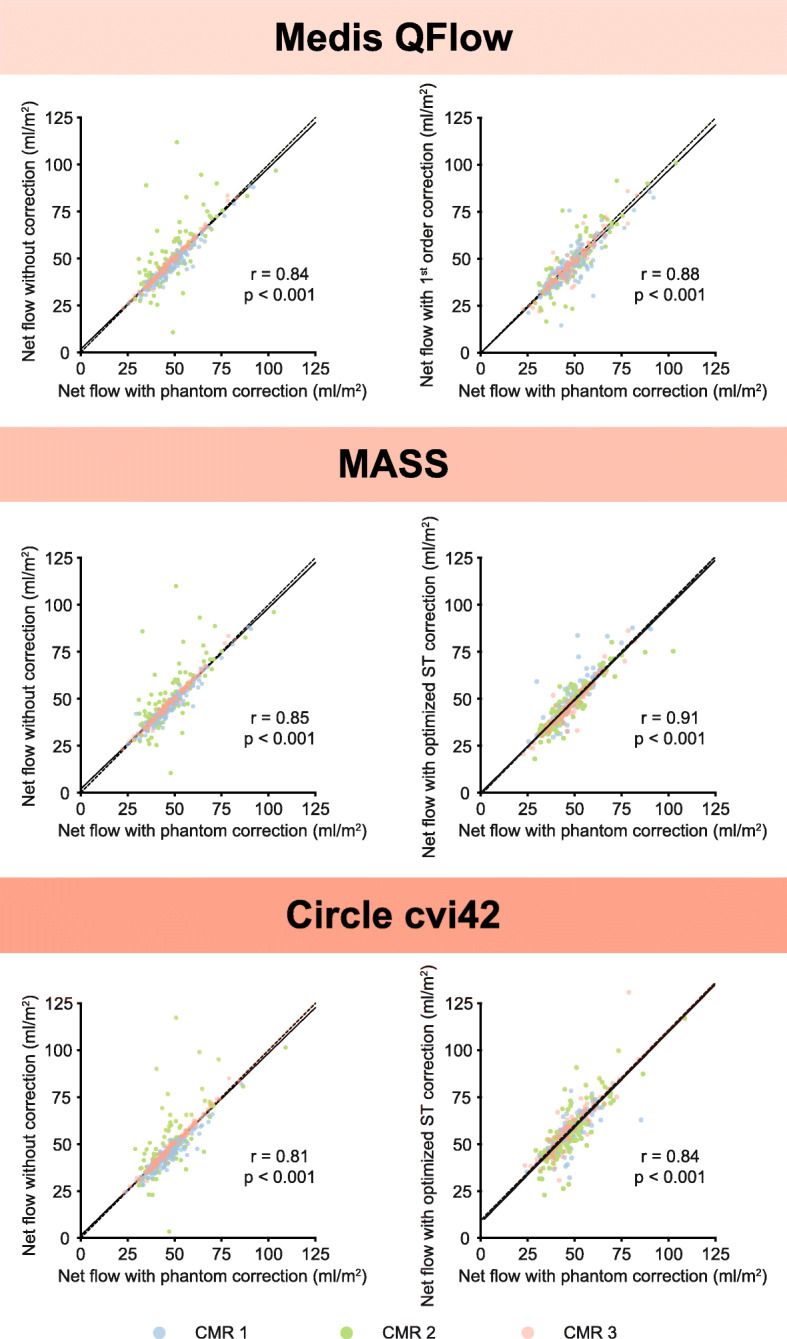
Scatterplots of net flow per body surface area (BSA) with phantom correction (x-axis) compared to uncorrected and optimized stationary tissue correction (y-axis) measured with Medis QFlow, Circle cvi42 and MASS software programs. Black line is least-squares linear regression line, dashed black line is x = y line. Pearson correlation coefficients are depicted with corresponding p-values

**Fig. 4:**
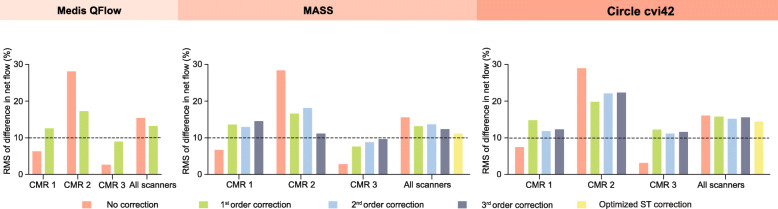
Root mean square (RMS) of the percentage difference in net flow between phantom correction and no correction, first, second, third order and optimized stationary tissue correction measured in three different software packages

The mean phantom corrected net flow was 48 ± 11 mL/m^2^ per heartbeat. There was a strong relationship between phantom corrected net flow and the net flow obtained using the optimized stationary tissue correction methods or the uncorrected net flow (Fig. 3 and Additional file 2). The net flow obtained using MASS software showed the strongest relationship with phantom corrected net flow (Pearson’s *r* = 0.91, *p* < 0.001). Overall, the mean difference and the absolute median difference in net flow did not ameliorate significantly with stationary tissue correction compared to no correction (Table 2). On the contrary, it deteriorated with most interpolation orders. Considering the percentage differences in net flow, uncorrected flow measurements resulted systemically in the smallest difference on CMR-1 and CMR-3 (Fig. 4). However, in spite of correction methods and software packages, on CMR-2 substantial over- and underestimation remained.

Consistent among the different software packages, stationary tissue correction resulted in significant more measurements with clinically significant (> 10%) differences in net flow compared to no correction. The number of measurements with clinically significant differences varied between 67 (19%) with optimized stationary tissue correction in MASS and 137 (40%) with first order Circle cvi42 stationary tissue correction. For uncorrected measurements, this varied between 62 (18%) with MASS and 80 (23%) with Circle cvi42 (Table 2). The frequency of clinically significant differences varied substantially between scanners (Additional file 3). For CMR-3, no correction was necessary, because in only 1 (1%) of 99 measurements there was a clinically significant difference in net flow of > 10% compared to phantom correction.

Correlations of Q_p_/Q_s_ ratios of uncorrected and stationary tissue corrected measurements compared to phantom corrected measurements are shown in Additional file 4. Both uncorrected and stationary tissue corrected flow measurements were more likely to overestimate than to underestimate Q_p_/Q_s_ ratios. When looking at a cut off of > 1.5 for having a hemodynamically significant shunt, no correction and stationary tissue correction resulted in more hemodynamically significant shunts compared to phantom corrected data. With phantom corrected measurements, 6 (Medis QFlow) to 7 (MASS and Circle cvi42) patients had a hemodynamically significant shunt, whereas with no correction or stationary tissue correction 10 (third order stationary tissue correction MASS) to 21 (first order stationary tissue correction Circle cvi42) patients had a hemodynamically significant shunt. Incorrect shunt estimation was most commonly caused by a relative overestimation of pulmonary flow compared to aortic flow.

### Regurgitation fraction

In general, regurgitation fraction was often overestimated with uncorrected and stationary tissue corrected measurements compared to phantom corrected measurements. Depending on software package and correction method, regurgitation was incorrectly classified up 37% of the assessed vessel with first order Circle cvi42 software (Table 3). Taking aortic and main pulmonary artery measurements together, second and third order and optimized stationary tissue correction could not categorize regurgitation better than no correction. Uncorrected regurgitation severity had the strongest agreement with phantom corrected regurgitation severity (weighed kappa coefficient between 0.64–0.92 depending on the software and vessel of interest), except for aortic regurgitation severity using third order stationary tissue correction from MASS and second order stationary tissue correction from Circle cvi42. Additional file 5 shows changes in aortic and pulmonary regurgitation severity comparing no correction and stationary tissue correction with phantom correction. Both stationary tissue and no correction tended to overestimate aortic regurgitation, whereas for pulmonary regurgitation there was no tendency towards over- or underestimation. On CMR-3, only in 2 (2%) of 99 measurements a change in regurgitation severity occurred using the uncorrected measurements.

**Table 3:** Regurgitation reclassification comparing no and stationary tissue correction methods to phantom correction

	Aortic valve (***n*** = 175)					Pulmonary valve (***n*** = 171)				
	≥1 category^a^	1 category^b^	≥2 categories^c^	Weighted kappa	95% CI	≥1 category^a^	1 category^b^	≥2 categories^c^	Weighted kappa	95% CI
**Medis QFlow**										
No correction	21 (12%)	19 (11%)	2 (1%)	0.69	0.55–0.83	16 (9%)	15 (9%)	1 (1%)	0.92	0.88–0.96
1st order correction	36 (21%)	31 (18%)	5 (3%)	0.55	0.40–0.69	19 (11%)	18 (11%)	1 (1%)	0.90	0.86–0.94
**MASS**										
No correction	21 (12%)	20 (11%)	1 (1%)	0.73	0.61–0.85	15 (9%)	14 (8%)	1 (1%)	0.92	0.88–0.96
Optimized ST correction	22 (13%)	19 (11%)	3 (2%)	0.69	0.55–0.83	18 (11%)	18 (11%)	0 (0%)	0.91	0.88–0.95
1st order correction	28 (16%)	22 (13%)	6 (3%)	0.62	0.49–0.76	22 (13%)	22 (13%)	0 (0%)	0.89	0.85–0.93
2nd order correction	24 (14%)	19 (11%)	5 (3%)	0.60	0.43–0.76	23 (13%)	23 (13%)	0 (0%)	0.89	0.85–0.93
3rd order correction	18 (10%)	15 (9%)	3 (2%)	0.73	0.59–0.87	23 (13%)	23 (13%)	0 (0%)	0.89	0.85–0.93
**Circle cvi42**										
No correction	29 (17%)	27 (15%)	2 (1%)	0.64	0.51–0.78	15 (9%)	14 (8%)	1 (1%)	0.92	0.88–0.96
Optimized ST correction	32 (18%)	27 (15%)	5 (3%)	0.61	0.48–0.74	35 (20%)	34 (20%)	1 (1%)	0.83	0.77–0.88
1st order correction	64 (37%)	55 (31%)	9 (5%)	0.41	0.28–0.53	26 (15%)	25 (15%)	1 (1%)	0.87	0.82–0.92
2nd order correction	24 (14%)	20 (11%)	4 (2%)	0.65	0.51–0.79	34 (20%)	32 (19%)	2 (1%)	0.83	0.77–0.88
3rd order correction	27 (15%)	23 (13%)	4 (2%)	0.63	0.50–0.77	31 (18%)	28 (16%)	3 (2%)	0.84	0.78–0.89

Values are presented as number (percentage)

*CI* Confidence interval, *ST* Stationary tissue

^a^ indicates number of studies in which regurgitation severity is reclassified with the different correction methods (no, 1st, 2nd or 3rd order) compared to phantom corrected measurements; ^b^ number of studies in which regurgitation severity shifted only one category (e.g. from mild to moderate). ^c^ number of studies in which regurgitation severity shifted with two categories or more (e.g. from none to moderate or mild to severe)

### Uni- and multivariate prediction of phase offset errors

Higher velocity offsets were significantly associated with higher maximum and average vessel size, VENC, maximum blood flow velocity, main pulmonary artery measurements and scanning performed on CMR-2. CMR-2 had the strongest association (β = 1.73, *p* < 0.001, 95% CI 1.37–2.09). Maximum velocity (m/s) (β = 0.26, *p* = 0.03, 95% CI 0.02–0.49) and CMR-2 (β = 1.69, *p* < 0.001, 95% CI 1.33–2.05) remained independently associated with velocity offset in multivariate analysis. In multivariate analysis, there was a trend for larger velocity offsets in the main pulmonary artery measurements (β = 0.31, *p* = 0.06) than the aortic measurements, however, this was not statistically different.

## Discussion

The main finding of this study was that phase offset errors have a large impact on PC based blood flow quantification in daily clinical practice. This resulted in clinically significant flow differences in 18–23% of the measurements depending on the analysis software program used and had a substantial impact on regurgitation classification. Surprisingly, stationary tissue correction independent of the order of interpolation (first, second and third) or even optimized per scanner and software program did not improve the accuracy of the flow measurements, but generally worsened the results. Results of our analyses acquired with three different software packages were comparable. However, we found a large difference in phase offset errors between scanners. In the best performing scanner, the phase offset errors were so small that no correction at all was necessary.

From a clinical point of view, the present study indicates that both uncorrected and stationary tissue corrected flow measurements could lead to clinically relevant differences in flow measurements and poor regurgitation indexing. Without correction the regurgitation severity classification changed in 9 to 17% of the cases depending on the software program used. However, this was 10 to 37% with stationary tissue correction. Incorrect regurgitation classification could potentially have therapeutic consequences in deciding whether valvular surgery is indicated. Therefore, neither strategy (no correction and stationary tissue correction) is shown to be ideal.

Inherent to PC imaging are phase offset errors caused by eddy-currents. Previous studies have already shown that phase offset errors can lead to clinically significant differences [3, 16–18]. Stationary tissue correction has been suggested as a reliable method to reduce phase offset errors [10] and is implemented into several commercial available post-processing software programs for CMR analysis (e.g. Medis QFlow (Leiden, The Netherlands), Circle cvi42 (Cardiovascular Imaging Inc., Calgary, Alberta, Canada), SyngoVia (Siemens Healthcare, Erlangen, Germany)). To our knowledge, this is the first study conducted in a large population in which stationary tissue correction was shown to worsen 2D flow measurement accuracy.

This study tested both linear and higher order spatial interpolation methods using three different software programs. Lankhaar et al. concluded for pulmonary artery flow measurements in their single center study that a first order surface fit, combined with regarding 25% of the pixels as stationary, minimized velocity offsets [3]. However, Hofman et al. showed in their multicenter study that the some GE systems require second order surface fit. Measurement errors after stationary tissue correction in cardiac output were expected to stay within an acceptable range for clinical application (0 ± 5%) when using this method [10]. The results of this study confirm that on our GE Healthcare scanners on certain scanners a second or third order surface fit was also preferred over first order surface fit. Nevertheless, on two of our three scanners no correction was better than any order of stationary tissue correction. On the third scanner substantial over- and underestimation remained despite different order correction methods. Only using the uncorrected measurements on CMR-3 resulted in cardiac output staying within this range.

Phase offset is not a problem that occurs linearly over the scan plane and, therefore, a first order correction is not perfect [6]. However, higher order corrections require a larger number of pixels with stationary tissue sufficiently scattered through the whole imaging plane. Because of insufficient available stationary tissue in the FOV and especially close to the vessel of interest, second, third or even higher order corrections are often unreliable. Therefore, it is important that during scanning the FOV and orientation of the scan plane should be optimized to include the maximum amount of stationary tissue possible.

The magnitude of phase offset errors varied among scanners, as has been reported in previous studies [9]. Maximum gradient amplitude and slew rate are known to influence eddy-currents and have proven to be individual predictors of phase offset errors [19]. These factors might have contributed to the variation of magnitude of phase offset errors we observed between our three scanners.

In this study, CMR-2 suffered more from eddy currents. Both with stationary tissue correction and no correction, measurement errors remained significant and, therefore, phantom scanning should be advised. Previously, poor flow quantifying performance of a similar type of scanner (Discovery MR450) has been reported [10]. However, variability in velocity offsets before and after stationary tissue correction on this scanner was larger in our study than in the previous study. We have no clear explanation for this observation. On the contrary, omitting phantom correction on CMR-3 would only lead to clinically relevant differences in 1% of the measurements. As also shown in this study, phase offset errors of one scanner differ substantially between patients, measurements, and slice orientation. Depending on the accuracy of the flow measurements that is acceptable for clinical decision making, we advise acquiring phantom scans in at least 30–50 patients to get familiar with your local CMR system.

The strength of this study is the number of patients and its consistent scanning protocol with direct phantom scanning to minimize the influence of temporal instability. Temporal stability was tested and only acquisitions with temporal stability between the in-vivo and phantom acquisition of ≤0.6 cm/s were included in the results of this study. In contrast to smaller previous studies, this study included 175 patients with 346 measurements. Furthermore, different stationary tissue order methods were compared using three different software programs.

### Limitations

This study evaluated only scans acquired with GE Healthcare CMR scanners. Therefore, our conclusions might not hold for scanners from a different vendor or other GE models. However, we have used the same sequence settings as Hofman et al. did and no other build-in correction methods were applied [10]. As the origin of velocity offset lies within the gradient system (slew rate and gradient amplitude) and its associated errors, theoretically no differences between vendors should be expected [10]. Hence, we expect that these results should also be valid for CMR systems from different vendors. Secondly, the static phantom correction, our reference standard, is not perfect because phase offset errors drifts over time. However, this drift is limited within a clinically acceptable range when scanning within the same imaging session on most systems [9]. Therefore, all phantoms were scanned directly after the CMR examination was finished. Furthermore, temporal stability of the phantom correction method was tested for each scan and studies with a large deviation of phase offset errors were excluded using the same method as Hofman et al. used [10]. Finally, in this study only a retrospective ECG gated PC sequence was used. In patients with an unstable heart rhythm, prospective ECG gating may provide more accurate results. However, with prospective ECG gating the velocity offset varies during the cardiac cycle depending on the timing after the sequence starts running [7]. This studies’ stationary tissue correction method assumes a constant velocity offset during the cardiac cycle and this method should, therefore, be validated on prospective ECG gated sequences where a correction per cardiac phase might be more appropriate.

## Conclusion

Phase offset errors had a significant impact on PC CMR based flow quantification and regurgitation assessment and varied greatly between scanners. Unexpectedly, background phase correction using stationary tissue correction worsened accuracy compared to no correction. Similar results were obtained independent of spatial order of interpolation and type of software used. Large clinically significant deviations in net flow and regurgitation severity index were present after stationary tissue correction. These results are solely based on images from GE Healthcare scanners and future research needs to investigate whether these same conclusions hold for other vendors as well. In general, careful assessment of phase offset errors at each individual scanner is essential to determine whether routine use of phantom correction is necessary.

## Supplementary information

**Additional file 1:** Characteristics of the three CMR systems.

**Additional file 2:** Correlations of net flow with phantom correction compared to stationary tissue correction.

**Additional file 3:** Clinically significant differences (> 10%) in net flow per scanner.

**Additional file 4:** Correlations of Qp/Qs ratios with phantom correction compared to uncorrected and stationary tissue correction.

**Additional file 5:** Change in aortic and pulmonary regurgitation severity indexing.

## Data Availability

The datasets used and/or analyzed supporting the conclusions of the article are available from the corresponding author on reasonable request.

## Abbreviations

BSA: Body surface area
CMR: Cardiovascular magnetic resonance
ECG: Electrocardiogram
EDV: End-diastolic volume
EF: Ejection fraction
ESV: End-systolic volume
FOV: Field-of-view
LV: Left ventricle/left ventricular
PC: Phase contrast
RV: Right ventricle/right ventricular
VENC: Velocity encoded
