# Supplementary material for: The clinical impact of phase offset errors and different correction methods in cardiovascular magnetic resonance phase contrast imaging: a multi-scanner study
Source: J Cardiovasc Magn Reson. 2020 Sep 17;22:68. doi: 10.1186/s12968-020-00659-3 (PMC7495876; doi:10.1186/s12968-020-00659-3)
Supplement: Supplementary file 1 — Additional file 1: Characteristics of the three CMR systems. [file 12968_2020_659_MOESM1_ESM.docx]

| **Additional file 1: Characteristics of the three CMR systems** | | | |
| --- | --- | --- | --- |
|  | **Signa Artist  (CMR-1)** | **Discovery MR450  (CMR-2)** | **Signa Explorer  (CMR-3)** |
| Software level | DV26.0 | DV25.0 | SV25.0 |
| Bore size (cm) | 70 | 60 | 60 |
| Maximum gradient amplitude (mT/m) | 44 | 50 | 33 |
| Slew rate (T/s/m) | 200 | 200 | 120 |
