# Supplementary figures and images for: The clinical impact of phase offset errors and different correction methods in cardiovascular magnetic resonance phase contrast imaging: a multi-scanner study
Source: J Cardiovasc Magn Reson. 2020 Sep 17;22:68. doi: 10.1186/s12968-020-00659-3 (PMC7495876; doi:10.1186/s12968-020-00659-3)

# MASS

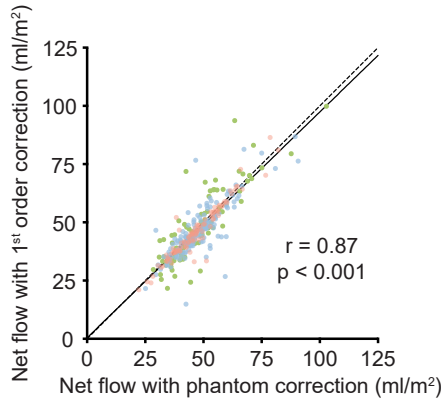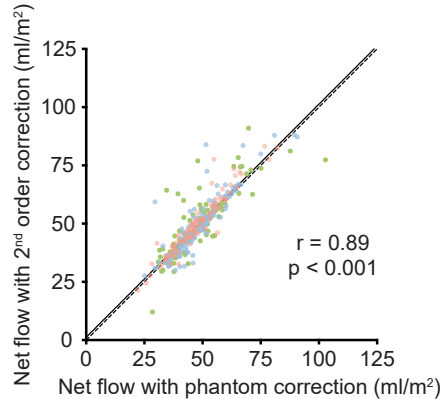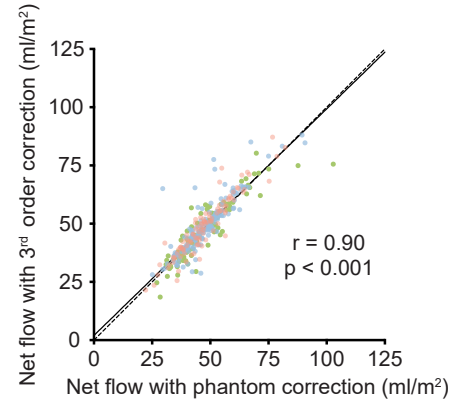

# Circle cvi42

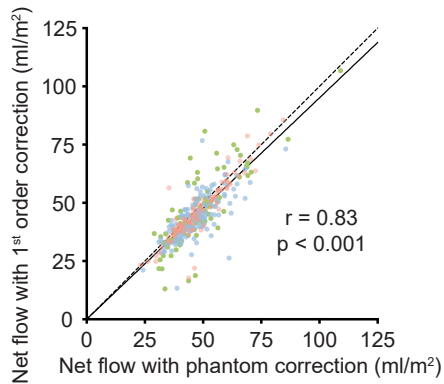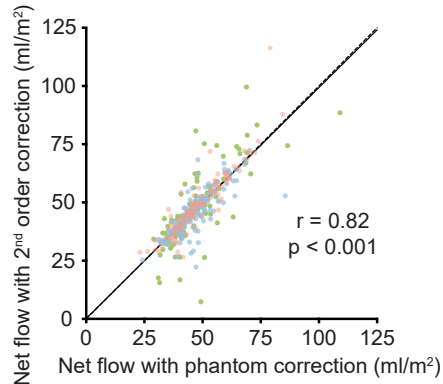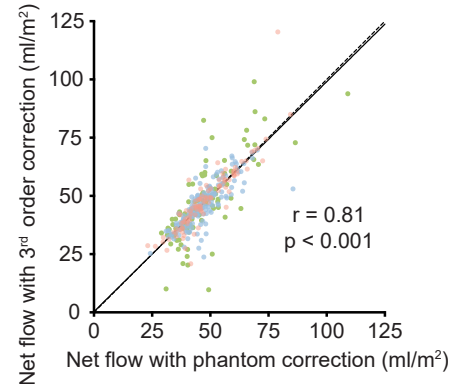

Supplement: Supplementary file 2 — Additional file 2: Correlations of net flow with phantom correction compared to stationary tissue correction. [file 12968_2020_659_MOESM2_ESM.pdf]

# Medis QFlow

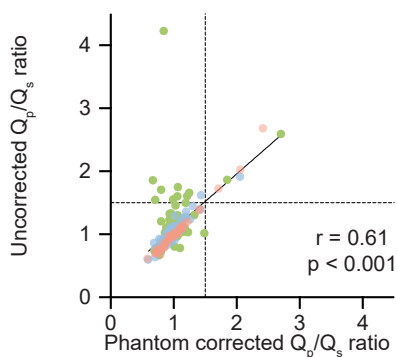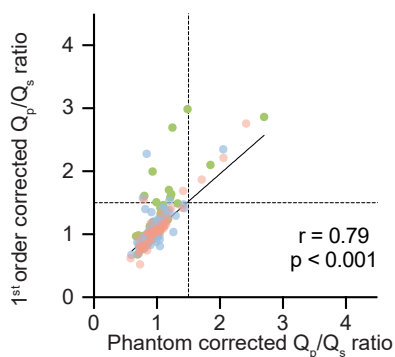

# MASS

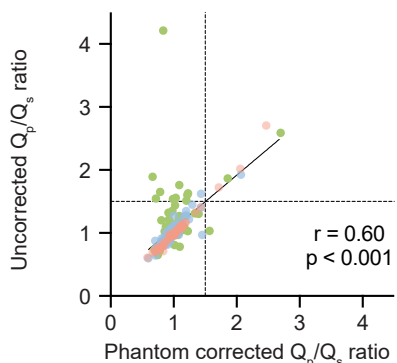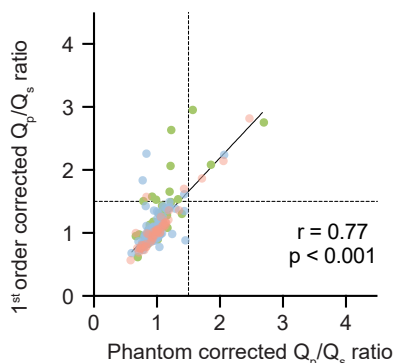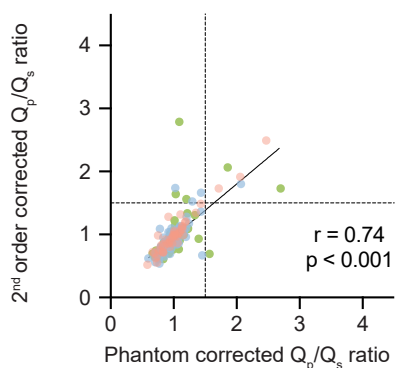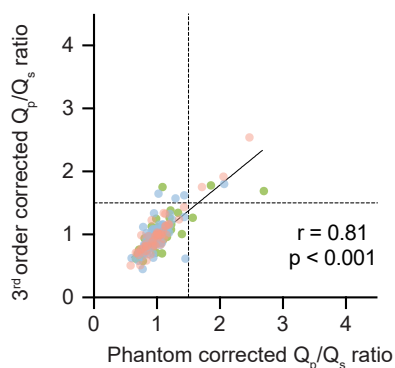

# Circle cvi42

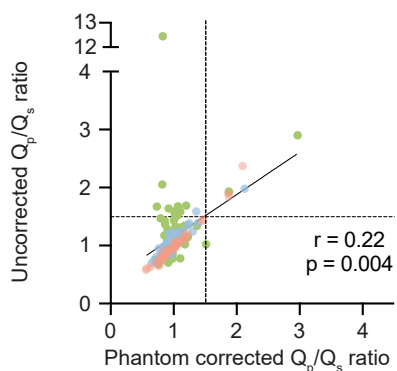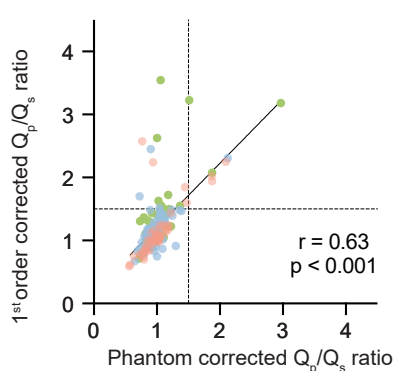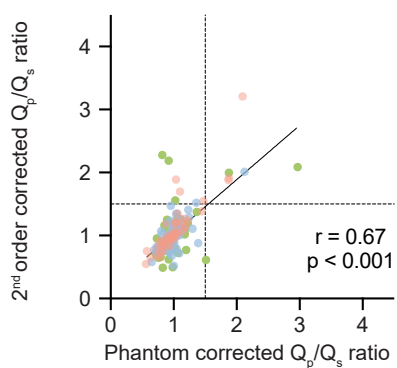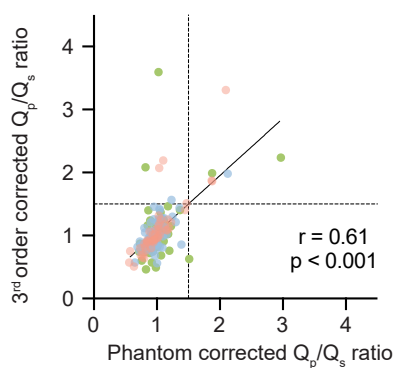

● CMR 1 ● CMR 2 ● CMR 3

Supplement: Supplementary file 4 — Additional file 4: Correlations of Qp/Qs ratios with phantom correction compared to uncorrected and stationary tissue correction. [file 12968_2020_659_MOESM4_ESM.pdf]
