# Supplementary material for: The clinical impact of phase offset errors and different correction methods in cardiovascular magnetic resonance phase contrast imaging: a multi-scanner study
Source: J Cardiovasc Magn Reson. 2020 Sep 17;22:68. doi: 10.1186/s12968-020-00659-3 (PMC7495876; doi:10.1186/s12968-020-00659-3)
Supplement: Supplementary file 3 — Additional file 3: Clinically significant differences (> 10%) in net flow per scanner. [file 12968_2020_659_MOESM3_ESM.docx]

| **Additional file 3: Clinically significant differences (>10%) in net flow per scanner** | | | | |  |
| --- | --- | --- | --- | --- | --- |
|  | **CMR-1 (n=152)** | **CMR-2 (n=95)** | **CMR-3 (n=99)** | **Total  (n=346)** | **P-values*** |
| **Medis QFlow** | | | | |  |
| No correction | 18 (12%) | 46 (48%) | 1 (1%) | 65 (19%) | <0.001 |
| 1^st^ order correction | 43 (28%) | 45 (47%) | 15 (15%) | 103 (30%) | <0.001 |
| **MASS** | | | | |  |
| No correction | 14 (9%) | 47 (49%) | 1 (1%) | 62 (18%) | <0.001 |
| 1^st^ order correction | 40 (26%) | 43 (45%) | 10 (10%) | 93 (27%) | <0.001 |
| 2^nd^ order correction | 27 (18%) | 35 (37%) | 12 (12%) | 74 (21%) | <0.001 |
| 3^rd^ order correction | 29 (19%) | 30 (32%) | 20 (20%) | 79 (23%) | 0.057 |
| **Circle cvi42** | | | | |  |
| No correction | 27 (18%) | 52 (55%) | 1 (1%) | 80 (23%) | <0.001 |
| 1^st^ order correction | 69 (45%) | 49 (52%) | 19 (19%) | 137 (40%) | <0.001 |
| 2^nd^ order correction | 36 (24%) | 51 (54%) | 20 (20%) | 107 (31%) | <0.001 |
| 3^rd^ order correction | 46 (30%) | 51 (54%) | 18 (18%) | 115 (33%) | <0.001 |
| Values are presented as number (percentage).  * p-values were calculated with a Chi-square test | | | | |  |
