# Supplementary material for: The clinical impact of phase offset errors and different correction methods in cardiovascular magnetic resonance phase contrast imaging: a multi-scanner study
Source: J Cardiovasc Magn Reson. 2020 Sep 17;22:68. doi: 10.1186/s12968-020-00659-3 (PMC7495876; doi:10.1186/s12968-020-00659-3)
Supplement: Supplementary file 5 — Additional file 5: Change in aortic and pulmonary regurgitation severity indexing. [file 12968_2020_659_MOESM5_ESM.pdf]

Additional file 5: Change in aortic and pulmonary regurgitation severity indexing

Medis QFlow

No correction \ Phantom correction	No	Mild	Moderate	Severe	
No	238	2	0	0	240
Mild	17	23	4	1	45
Moderate	1	3	18	6	28
Severe	1	0	2	30	33
	257	28	24	37	

First order stationary tissue correction \ Phantom correction	No	Mild	Moderate	Severe	
No	223	2	0	0	225
Mild	31	21	5	1	58
Moderate	1	3	17	6	27
Severe	2	2	2	30	36
	257	28	24	37	

MASS

No correction \ Phantom correction	No	Mild	Moderate	Severe	
No	236	15	1	1	252
Mild	5	24	5	1	35
Moderate	0	2	18	7	27
Severe	0	0	0	32	32
	241	41	24	40	

First order stationary tissue correction \ Phantom correction	No	Mild	Moderate	Severe	
No	223	7	1	1	232
Mild	16	30	5	1	51
Moderate	0	2	14	10	26
Severe	2	2	4	29	37
	241	41	24	40	

Second order stationary tissue correction \ Phantom correction	No	Mild	Moderate	Severe	
No	231	16	2	1	250
Mild	9	21	5	1	36
Moderate	0	4	14	5	23
Severe	1	0	3	33	37
	241	41	24	40	

Third order stationary tissue correction \ Phantom correction	No	Mild	Moderate	Severe	
No	231	14	1	1	247
Mild	9	22	5	0	36
Moderate	1	5	15	2	23
Severe	0	0	3	37	40
	241	41	24	40	

Circle cvi42

No correction \ Phantom correction	No	Mild	Moderate	Severe	
No	227	4	0	0	231
Mild	23	25	3	1	52
Moderate	1	3	21	5	30
Severe	1	0	3	29	33
	252	32	27	35	

First order stationary tissue correction \ Phantom correction	No	Mild	Moderate	Severe	
No	189	1	0	0	190
Mild	57	22	4	0	83
Moderate	2	5	16	6	29
Severe	4	4	7	29	44
	252	32	27	35	

Second order stationary tissue correction \ Phantom correction	No	Mild	Moderate	Severe	
No	223	10	0	0	233
Mild	24	18	5	0	47
Moderate	5	3	16	4	28
Severe	0	1	6	31	38
	252	32	27	35	

Third order stationary tissue correction \ Phantom correction	No	Mild	Moderate	Severe	
No	220	7	0	0	227
Mild	27	19	5	0	51
Moderate	4	4	17	3	28
Severe	1	2	5	32	40
	252	32	27	35	
